# Imaging dose and image quality of kilovoltage imaging implemented on a helical tomotherapy unit

**DOI:** 10.1016/j.zemedi.2024.12.003

**Published:** 2025-01-21

**Authors:** Eric D. Ehler, Parham Alaei

**Affiliations:** Department of Radiation Oncology, University of Minnesota, Minneapolis, MN, USA

**Keywords:** IGRT, imaging dose, ClearRT, CBCT

## Abstract

The purpose of this work was to evaluate the imaging dose for the Accuray Radixact ClearRT system. Low-contrast resolution and CT number consistency was evaluated as well. CTDI measurements were compared to vendor supplied values, and similar measurements were done on a Varian TrueBeam kV cone-beam CT (CBCT) and a Philips Big Bore CT scanner. In-field imaging doses were measured using various protocols in an anthropomorphic phantom, while out-of-field doses were measured 10 cm from the imaging field edge. Comparison of the CTDI and the in-field doses showed considerable disagreement when the patient anatomy size was not congruent with the size of the CTDI phantom. While that is an expected outcome, this work provides an estimate of the differences for a variety of ClearRT protocols when this situation arises. The CNR was measured for all combinations of ClearRT settings for comparisons within the system, as well as for a comparison with a CBCT and fan-beam CT system. The CNR and dose information provided in this work can be used to aid in selecting a ClearRT imaging protocol. The CT number stability was tracked over 27 months; two instances where the CT number constancy exceeded tolerance were observed after service.

## Introduction

ClearRT is an optional imaging modality on the Accuray Radixact helical tomotherapy systems (Accuray, Sunnyvale, CA). This imaging modality includes a kilovoltage (kV) X-ray tube with a peak voltage of up to 140 kV and a flat panel detector array of Csi: Tl orthogonal to the megavoltage beamline. The benefits of this imaging modality include better soft tissue contrast than megavoltage cone-beam CT (MVCT) utilized in helical tomotherapy units, as well as faster imaging times.

Numerous studies have been conducted on the imaging dose and image quality of kilovoltage X-ray units added to radiation therapy delivery systems. For example, two AAPM Task Group reports have extensively discussed the imaging dose from various systems [1], [2]. There are multiple publications on imaging dose magnitudes from kilovoltage imaging [3], [4], [5], [6]. There have also been reports on the imaging dose and image quality from MVCT imaging on Tomotherapy/Radixact units [6], [7], [8], [9]; however, there is limited information published on ClearRT imaging dose. Ferris et al. [10], [11] measured the kV imaging dose from a similar unit but only for kV radiographs. Two previous studies [12], [13] performed an image quality assessment of ClearRT and compared that to cone beam CT (CBCT) and a fan-beam CT system; both studies investigated a subset of all possible image acquisition parameters for ClearRT. This work includes measurements of the contrast-to-noise ratio (CNR) for all possible ClearRT image acquisition setting combinations. Additionally, new imaging protocol options have been released for ClearRT since the publication of previous studies and have been included in this study. The imaging unit can scan volumes of up to 1350 mm in length with three fields of view (FOV) of 270, 440, and 500 mm. The larger fields of view are obtained by offsetting the detector array. ClearRT employs a total of 99 imaging protocols (when fully implemented) obtained by a combination of user-defined parameters including “Anatomy”, “Body Size”, “Mode”, and “FOV”. These parameters determine the peak energy (kV), current (mA), filtration type, and imaging pitch. Additionally, the helical nature of ClearRT allows the length of the imaged volume to be defined by the user. All of these parameters affect the imaging dose and image quality. The protocols are summarized in Supplementary Table 1.

The goals of the present work were to: 1) Determine the CT Dose Index (CTDI) of ClearRT and compare it to the vendor-supplied values and another CBCT system, as well as a fan beam CT, 2) Measure the imaging dose from this unit inside and outside of an anthropomorphic phantom for use in patient and scatter dose estimations and comparing it to other systems, 3) Evaluate the CNR for all protocols employed, and 4) Describe the stability of the CT number values over time for the system.

For all of the above, comparison measurements were made on a C-arm linac cone beam CT (CBCT) unit and a diagnostic CT scanner commonly employed in radiation therapy settings. The data presented here are useful for clinical decision-making regarding imaging protocol selection for users of these units. CNR was chosen as the image quality parameter to consider because of its relationship with the imaging dose. Moreover, a high CNR is often desirable for soft tissue registration. Finally, because ClearRT images can potentially be used for treatment planning and/or adaptive planning, the CT number stability over time is presented. An unstable relationship of CT number to density can result in loss of dosimetric calculation accuracy [14].

The CTDI and CNR data tabulated in this paper can assist clinicians in choosing the appropriate imaging protocols for patients, minimizing the imaging dose while maintaining image quality. The CTDI, however, is not equivalent to patient dose and dose measurements in an anthropomorphic phantom provide the necessary data for estimating patient doses. Similarly, measuring out-of-field dose for ClearRT and comparing it to that from other imaging modalities provides the necessary data when out-of-field dose is of importance (e.g. for pregnant patients, implanted devices, etc.), especially when deciding between different treatment modalities, i.e. tomotherapy vs. C-arm linacs.

## Methods

### Beam output measurements

A common method of characterizing a CT scanner’s radiation dose output is by determining the CTDI as defined by Shope et al. [15] The CTDI was originally defined for axial scanning without couch movement; however, there are limitations in using it for both helical CT and CBCT scans. These limitations have previously been addressed, leading to updated forms of beam output measurements, such as the IAEA Human Health Report No. 5 [16] and AAPM TG-111 report [17]. For the purpose of this study, we chose to use the traditional CTDI, as it is still commonly used for diagnostic CT scanner units, and since our goal was to compare the ClearRT imaging dose to other modalities.

The kV CT images of the Radixact system are acquired helically, similar to modern fan-beam CT scanners, and there are no options for axial scanning available to users. Hence, evaluation was performed using a modified CTDI_vol_ measurement method proposed by Leon et al. [18] and compared to similar measurements performed on a Varian TrueBeam kV CBCT (Varian Medical Systems, Palo Alto, CA) and a Philips Brilliance Big Bore CT simulator (Philips Medical Systems, Cleveland, OH). In this method, a helical CTDI formalism is employed to determine the CTDI_vol_, ignoring the length of the 100 mm pencil chamber, slice thickness, number of slices, and pitch. Leon et al. showed that their method achieved reasonable agreement with that obtained by using the traditional method. In this study, we compared our CTDI measurements with those provided by Accuray. It should be noted that vendor-provided values have been measured with axial scanning, which is not clinically available [19].

All measurements were performed in either a standard cylindrical 16 cm diameter head or 32 cm diameter body CTDI phantom using a 100 mm pencil chamber (RaySafe X2, Fluke Biomedical, Cleveland, OH). For each protocol, measurements were performed at the center and at four peripheral locations, and the CTDI_vol_ was computed using Eq. (1):

(1) $${\mathrm{CTDI}}_{\mathrm{vol}}^{H}\left(mGy\right)=\left(\frac{1}{3}\times M_{H}^{\mathrm{center}}+\frac{2}{3}\times \overset{¯}{M_{H}^{\mathrm{peripheral}}}\right)$$

where M_H_ is the meter reading (in mGy) obtained from the chamber readings (in mR) for each position. $\overset{¯}{M_{H}^{\mathrm{peripheral}}}$ is the mean of four measurements at the periphery of the phantom. The length of the imaged volume for these measurements was set to match the CBCT reconstructed length of approximately 18 cm. This length is longer than the 16 cm length of the phantom and the 10 cm length of the pencil chamber, but was selected because the purpose of these measurements was to compare the systems. This is also longer than that used by Leon et al., who limited the scan length to that of the chamber air volume. Hereafter, ${\mathrm{CTDI}}_{\mathrm{vol}}^{H}$ will be referred to as “CTDI vol”.

### Imaging dose measurements

#### In-field imaging dose

**Figure 1:**
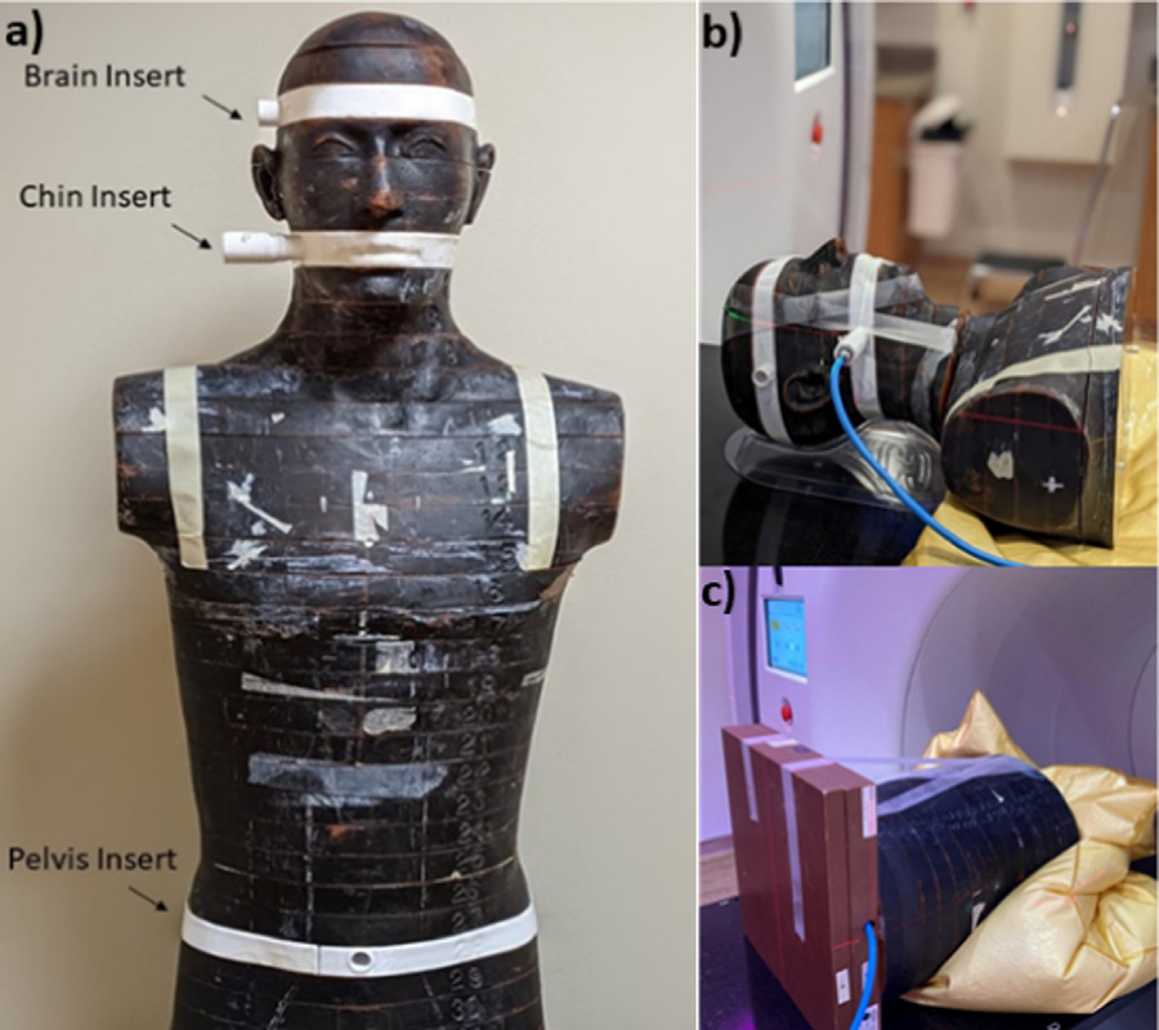
**a)** Anthropomorphic phantom with 3D printed inserts for the head, chin, and pelvis. **b)** Anthropomorphic phantom set up for ionization chamber measurements in the chin 3D printed insert. **c)** Out-of-field measurement setup for imaging of the pelvis region.

The imaging dose within the scan volume for a range of protocols was measured in an anthropomorphic phantom to simulate in-vivo imaging dose; these measurements will hereafter be referred to as “in-field”. To do this, three slices (in the brain, chin, and pelvis) of a Rando Anthropomorphic phantom (RSD, Long Beach, CA) were replaced by 3D-printed ones, each with an insert for a 0.6 cc Farmer-type chamber (Fig. 1a). The inserts were printed with 2.4 mm thick PET-G plastic. One surface of the insert was left open and filled with a mixture of water, glycerin, and gelatin to simulate soft tissue. The 3D printed slabs allowed a Farmer-type ionization chamber to be inserted with the sensitive volume at the center of the slab.

The “in-field” measurements were performed with a NIST traceable Farmer-type ionization chamber (PTW N30013, PTW GmbH, Freiburg, Germany). Similar measurements were performed using the Varian TrueBeam CBCT and Philips CT scanners. Fig. 1b shows an example of the phantom setup for imaging-dose measurements in the chin.

A comparison of “in-field” measurements with vendor-supplied CTDIvol was performed. A combination of imaging parameters were selected at the head, chin, and pelvis areas to provide a variety of clinically reasonable ClearRT scan protocols. A secondary goal of the comparison was to determine the difference in dose when the patient anatomy is not congruent with the phantom used for the determination of CTDI. This can happen when the imaging area encompasses two regions of anatomy with distinct thickness differences, such as the thorax and neck. For example, for nodal breast treatments a situation might arise where the image guidance region could encompass both the thorax and the neck. It is pertinent to highlight that for CTDIvol measurements, a 16 cm diameter phantom is used for head protocols and a 32 cm diameter phantom for other protocols.

#### Out of field imaging dose

To evaluate the out-of-field dose from imaging, the same anthropomorphic phantom was utilized, and the ion chamber was placed inside solid water slabs positioned such that the measurement point was 10 cm from the edge of the imaging field. Fig. 1c shows an example of the phantom setup for out-of-field imaging dose measurements in the pelvis. The same Farmer-type chamber was used in the out-of-field measurements as for the “in-field” measurements.

### Contrast noise ratio

CNR evaluations were performed using the CatPhan low-contrast phantom module (CTP 515) within the CatPhan 504 phantom (The Phantom Laboratory, Greenwich, NY) and Radia Module of the RIT software package (Radiological Imaging Technology, Colorado Springs, CO). A one cm diameter region of interest (ROI) was sampled within the largest 1% low-contrast insert and compared to a one cm diameter ROI just outside of the low-contrast insert (background), as described in other works (Gardner et al. [20] & Stock et al. [21]); The CNR was defined as:

(2) $$CNR=\;\frac{\left|m_{insert}+m_{background}\right|}{\sqrt{{\sigma^{2}}_{insert}+{\sigma^{2}}_{background}}}$$

where $m_{insert}$ and $m_{background}$ refer to the mean CT pixel value, and $\sigma_{insert}$ and $\sigma_{background}$ are the standard deviations for the low-contrast insert and background, respectively. CNR was measured for each combination of image parameter settings for Clear RT, as well as for the Varian CBCT and select Philips CT protocols. For Philips CT and Varian CBCT, comparison with Clear RT was provided for the most similar “Anatomy” scan protocol available.

### CT number stability

CT number stability was measured using a cylindrical (300 mm diameter) water equivalent phantom (commonly referred to as the “cheese phantom”), which is made from Solid Water HE (Sun Nuclear, Melbourne, FL). A CT number to density ratio was measured according to the Accuray user documentation prior to September 2022. This is important because in that method it was only recommended to measure the CT numbers for “Fine” scan modes. Vendor documentation published after September 2022 provides a method to measure CT numbers for both “Normal” and “Fine” scan modes. No method to measure CT number was provided at the start of the tracking for 270 mm FOV protocols. Additionally, the vendor recommends an additional weekly CT number calibration procedure that only tracks the CT numbers for air and the Solid Water HE phantom material without other tissue plugs (i.e., lung, cortical bone, etc.). This procedure was always performed just prior to monthly (and post-service) CT number to density measurements.

CT numbers were sampled over a range of tissue-equivalent density materials. Two different lung density materials, an inner bone, and three different density bone materials (variable calcium concentrations), as well as water and air, were measured. Measurements were performed monthly as part of the regular machine quality control program with the “Fine” scan mode and 440 mm FOV for the Head, Thorax, and Pelvis “Anatomy” protocols. Approximately one year after installation, aluminum was also measured in a separate scan to reduce streaking artifacts between high-density materials. Analysis was performed using RIT software.

## Results

### CTDI determination

The results of the CTDI_vol_ measurements for a number of available protocols on the Radixact unit are tabulated in Table 1 along with the vendor-provided values. The Accuray-provided values were measured axially, which is not a mode available to the users. The CTDI_vol_ values changed with “Body Size” setting for each “Anatomy” region, as expected.

**Table 1:** Measured vs provided CTDIvol for several ClearRT protocols.

**Phantom**	**Anatomy**	**Mode**	**Body Size**	**FOV (mm)**	**kVp**	**mA**	**Measured CTDI_vol_ (cGy)**	**Provided CTDI_vol_ (cGy)**	**% Difference**
Head	Head	Fine	Small	270	100	80	0.58	0.52	11.5%
			Medium			125	0.91	0.81	12.3%
			Large			160	1.17	1.04	12.5%
		Normal	Small			80	0.36	0.33	9.1%
			Medium			125	0.57	0.52	9.6%
			Large			160	0.73	0.66	10.6%
		Coarse	Small			80	0.18	0.17	5.9%
			Medium			125	0.29	0.27	7.4%
			Large			160	0.37	0.34	8.8%

**Body**	**Thorax**	**Fine**	**Small**	**440**	**120**	**80**	**0.85**	**0.85**	**0.0%**
			**Medium**			**125**	**1.32**	**1.33**	**-0.8%**
			**Large**			**160**	**1.67**	**1.70**	**-1.8%**
			**XL**			**200**	**2.12**	**2.13**	**-0.5%**
		**Normal**	**Small**			**80**	**0.63**	**0.63**	**0.0%**
			**Medium**			**125**	**0.98**	**0.98**	**0.0%**
			**Large**			**160**	**1.26**	**1.25**	**0.8%**
			**XL**			**200**	**1.55**	**1.56**	**-0.6%**
		**Coarse**	**Small**			**80**	**0.45**	**0.45**	**0.0%**
			**Medium**			**125**	**0.70**	**0.70**	**0.0%**
			**Large**			**160**	**0.90**	**0.90**	**0.0%**
			**XL**			**200**	**1.11**	**1.13**	**-1.8%**
Body	Pelvis	Fine	Small	440	140	80	1.22	1.23	-0.8%
			Medium			125	1.89	1.92	-1.6%
			Large			160	2.38	2.46	-3.3%
			XL			200	3.11	3.08	1.0%
		Normal	Small			80	0.93	0.89	4.5%
			Medium			125	1.38	1.39	-0.7%
			Large			160	1.80	1.78	1.1%
			XL			200	2.33	2.23	4.5%
		Coarse	Small			80	0.65	0.64	1.6%
			Medium			125	1.01	1.00	1.0%
			Large			160	1.29	1.28	0.8%
			XL			200	1.67	1.60	4.4%
**Body**	**Whole Body**	**Fine**	**Small**	**440**	**120**	**80**	**0.84**	**0.85**	**-1.2%**
			**Medium**			**100**	**1.05**	**1.06**	**-0.9%**
			**Large**			**125**	**1.30**	**1.33**	**-2.3%**
		**Normal**	**Small**			**80**	**0.62**	**0.63**	**-1.6%**
			**Medium**			**100**	**0.78**	**0.78**	**0.0%**
			**Large**			**125**	**0.98**	**0.98**	**0.0%**
		**Coarse**	**Small**			**80**	**0.45**	**0.45**	**0.0%**
			**Medium**			**100**	**0.56**	**0.56**	**0.0%**
			**Large**			**125**	**0.70**	**0.70**	**0.0%**

The results of similar measurements for Varian TrueBeam and Philips CT are also tabulated in Tables 2 and 3. For both units, the provided or displayed values and their percentage differences with the measured values are listed.

**Table 2:** Measured vs. provided CTDIvol for Varian TrueBeam CBCT protocols.

**Phantom**	**Protocol**	**kVp**	**mA**	**mAs**	**Measured CTDI_vol_ (cGy)**	**Provided CTDI_vol_ (cGy)**	**% Difference**
Head	Image Gently	80	20	100	0.11	0.094	17.0%
	Head	100	15	150	0.36	0.30	12.5%
Body	Thorax	125	60	270	0.44	0.40	10.0%
	Spotlight	125	60	750	1.47	1.20	19.5%
	Pelvis	125	60	1080	1.83	1.60	14.4%
	Pelvis Large	140	75	1688	3.84	3.70	3.8%

**Table 3:** Measured vs. provided CTDI values for several clinical imaging protocols for Philips Big Bore CT scanner

**Phantom**	**Protocol**	**kVp**	**mA**	**mAs**	**FOV (mm)**	**Measured CTDI_vol_ (cGy)**	**Provided CTDI_vol_ (cGy)**	**% Difference**
Head	OncoHead, Brain 3mm	120	40	500	600	5.16	5.31	-2.8%
		100	40	500	600	3.15	3.17	-0.6%
Body	OncoBody, Abd 3mm	120	50	463/Slice	500	2.64	2.41	9.5%
	OncoPed, Chest	100	30	150	350	0.53	0.52	1.9%

Comparing the measured CTDIvol values among comparable protocols on Radixact ClearRT and Varian TrueBeam CBCT shows similar values between “Head”-“Small” protocol on ClearRT and “Head” protocol on TrueBeam CBCT. The ClearRT CTDIvol values are greater than CBCT when “Head”-“Medium” or “Head”-“Large” options are utilized. For the Thorax protocols, CTDIvol values were consistently larger for ClearRT than for CBCT. On the other hand, ClearRT “Pelvis”-“Small” and “Pelvis”-“Medium” have lower CTDIvol values than CBCT “Pelvis” while ClearRT “Pelvis”-“Large” is comparable values to CBCT “Pelvis”. The CBCT “Pelvis Large” protocol had the highest CTDI value.

### Imaging dose measurements

#### In-field dose

Table 4 shows the dose measured in an anthropomorphic phantom within the imaging field for select protocols. For some anatomic settings, other parameters, such as “Size”, “Mode”, and “FOV”, were varied.

**Table 4:** Measured imaging doses “in-field” for ClearRT. CBCT, and CT along with CTDIvol data for comparison

**Body Site of Measurement**	**Modality**	**Anatomic Mode**	**Size**	**Mode**	**FoV (mm)**	**Measured Dose (cGy)**	**Provided CTDIvol (cGy)**	**% Difference**
Head	ClearRT	Head	Large	Fine	440	1.8	2.09	-13.9%
					270	0.9	1.04	-13.5%
		**Whole Body**	**Large**	**Fine**	**440**	**2.3**	**1.33**	**72.9%**
					**270**	**1.3**	**1.03**	**26.2%**
			**Medium**	**Normal**	**440**	**1.4**	**0.78**	**79.5%**
	CBCT	Head				0.3	0.30	0.0%
	CT _(100 kV)_	OncoHead, Brain 3mm				2.3	3.17	-27.4%
	CT _(120 kV)_					3.3	5.16	-36.0%

Pelvis	ClearRT	Pelvis	XL	Fine	440	3.6	3.08	16.9%
				Normal		2.7	2.23	21.1%
		Thorax	XL	Fine	440	2.4	2.13	12.7%
				Normal		1.8	1.56	15.4%
		Whole Body	Large	Fine	440	1.5	1.33	12.8%
				Normal		1.1	0.98	12.2%
	CBCT	Thorax				0.6	0.40	50.0%
	CBCT	Pelvis				2.4	1.60	50.0%
	CBCT	Pelvis Large				5.2	3.70	40.5%
	CT	OncoBody, Abd 3mm				1.6	2.41	-33.6%

Chin		Head	Large	Fine	440	2.3	2.09	10.0%
					270	1.1	1.04	5.8%
		**Thorax**	**XL**	**Fine**	**440**	**4.3**	**2.13**	**101.9%**
				Normal		3.4	1.56	**117.9%**
		**Whole Body**	**Medium**	**Normal**	**440**	**1.8**	0.78	**130.8%**
					**270**	**0.8**	**0.52**	**53.8%**
		**Whole Body**	**Large**	**Fine**	**440**	**2.8**	**1.33**	**110.5%**
					**270**	**1.5**	**1.03**	**45.6%**
	CBCT	Head				0.4	0.3	33.3%
	CBCT	**Thorax**				**1.2**	**0.4**	**200.0%**
	CT _(100 kV)_	OncoHead, Brain 3mm				2.9	3.17	-8.5%
	CT _(120 kV)_					4.9	5.31	-7.7%
	CT	**OncoPed, Chest**				**1.5**	**0.52**	**188.5%**

It can be seen in Table 4 that the measured ClearRT “in-field” doses agree with the CTDIvol measurements within +/- 20% when the CTDI phantom and anatomy size are similar. The shaded regions of the table (with considerably larger differences) are for situations when the provided CTDI phantom size is not congruent with that of the anatomy (e.g. CTDI measured with the body phantom but the anatomy is smaller as discussed in Section 2.2.1). This will be further elaborated on in the Discussion Section.

Table 4 also shows that of the “in-field” imaging dose among the three modalities, ClearRT has a higher imaging dose than CBCT for head and thorax imaging protocols. ClearRT pelvis protocols (at “Body Size” – “XL”) are similar to the CBCT “Pelvis” protocol; the CBCT “Pelvis Large” measured dose is about 2 times greater than both previously mentioned imaging protocols.

#### Out-of-field dose

The measurements of the out-of-field imaging dose at 10 cm from the imaging field edge are listed in Table 5. The data indicate that ClearRT imaging results in higher doses outside the imaging field for head, neck, and thorax imaging than both TrueBeam CBCT and Philips CT. It also shows that the out-of-field dose increases from “Fine” to “Normal” to “Coarse” settings.

**Table 5:** Out-of-field dose measurements for scans over the chin and pelvis regions

**Body Site**	**Modality**	**Anatomy**	**Size**	**Mode**	**FoV**	**Dose (mGy)**
Pelvis	ClearRT	Pelvis	XL	Fine	440	3.3
				Normal		4.5
				Coarse		6.8
		Thorax	XL	Fine	440	2.0
				Normal		2.6
				Coarse		3.8
		Whole Body	Large	Fine	440	1.3
				Normal		1.7
	CBCT	Thorax				0.6
		Pelvis				2.2
		Pelvis Large				4.9
	CT _(120kV)_					2.1

Chin	ClearRT	Head	Large	Fine	440	1.2
					270	0.6
		Whole Body	Large	Fine	440	1.7
					270	0.8
		Whole Body	Medium	Normal	440	1.5
					270	0.6
		Thorax	XL	Fine	440	2.6
				Normal		3.5
	CBCT	Head				0.2
	CT _(100 kV)_	Head				1.7
	CT _(120 kV)_					3.1

### Contrast noise ratio

**Figure 2:**
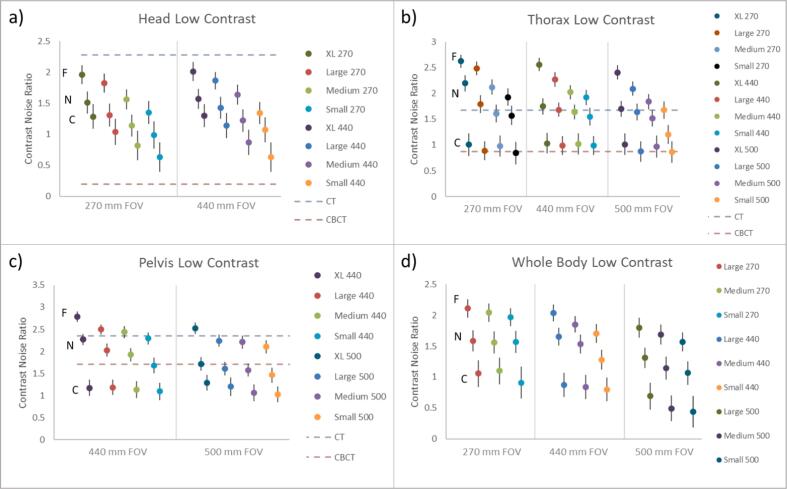
Low contrast detectability measured via CNR. Each body type protocol set is shown in panels a – d. “Body Size” is indicated by color and FOV is identified on the x-axis. On the left most data set of each body type subfigure, the location of the Fine (F), Normal (N), and Coarse (C) scan type is identified and this pattern is held for all subgroups (identified by color) to the right, i.e. the Fine scan type always showed the highest CNR and the Coarse scan type showed the lowest CNR.

The CNR for each scan parameter combination is shown in Fig. 2. Vertical bars indicate one standard deviation. The CNR for the Philips CT and Varian CBCT are indicated by dashed lines for comparison at the Head, Thorax, and Pelvis anatomy protocol sets. In general, the CNR for ClearRT “Head” scans is greater than CBCT and less than CT while for “Thorax” and “Pelvis” scans, the CNR exceeds both CBCT and CT for some protocols.

### CT number stability

**Figure 3:**
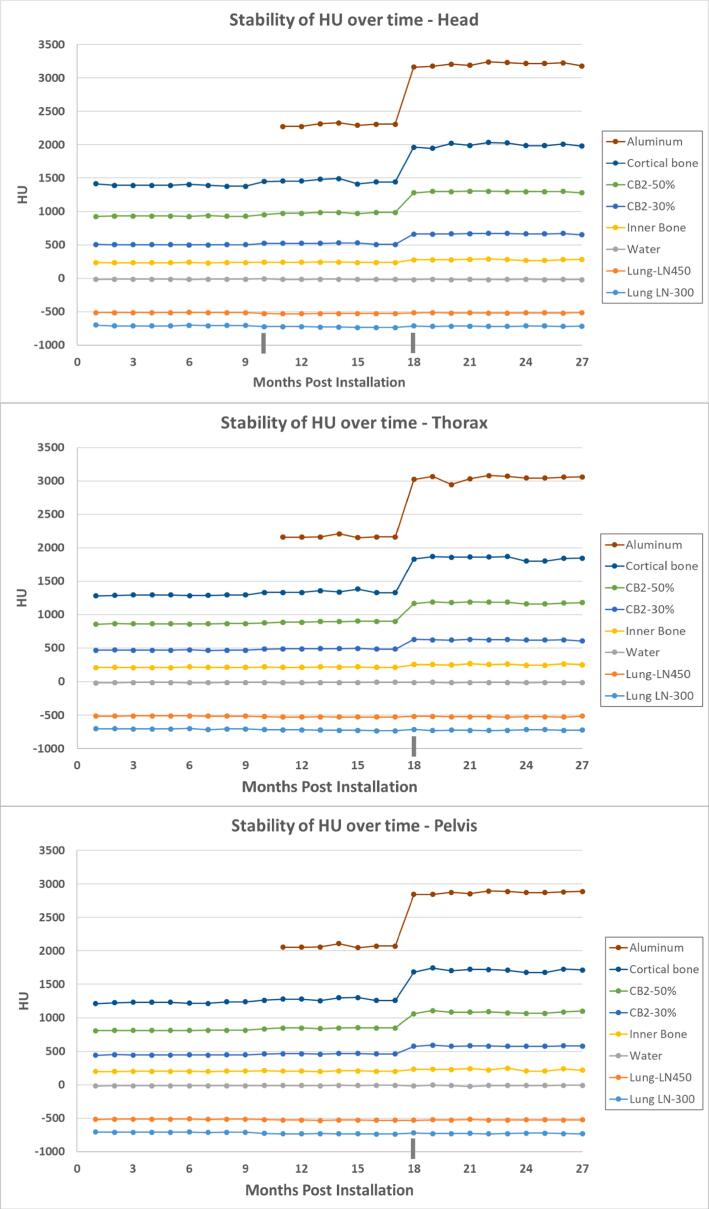
CT number stability over time for various tissue equivalent plugs for a) Head, b) Thorax, and c) Pelvis anatomic sites for Clear RT. The tick marks on the x-axis indicate times when the CT numbers exceeded the tolerance for dose calculation. A considerable change was observed for the “Head”, “Thorax”, and “Pelvis” protocols at month 18 which coincided with a software upgrade and power conditioner replacement. The only other adjustment was for the “Head” protocol at month 10 which coincided with a software update (see Discussion Section).

Fig. 3 shows CT number stability over several months for a variety of tissue simulating plugs. CT numbers were measured monthly, and the x-axis was numbered in months post-installation. The vertical line at month 10 indicates the cortical bone plug exceeding the tolerance for dose calculation of ± 50 for the “Head” scan; the tick mark at month 18 indicates a time when many of the high-density plugs exceeded the dose calculation CT number tolerance for “Head”, “Thorax”, and “Pelvis” scans. This sudden change happened after a software upgrade and power conditioner replacement.

## Discussion

Comparing the measured versus vendor-supplied CTDIvol values for ClearRT, the differences were within 5% except for the “Head” anatomy where differences of up to 13% were observed (Table 1). These differences were up to 20% for CBCT (Table 2) The source of the variation for the CBCT is unclear, but is probably related to the method and phantom employed by the vendor to determine CTDIs. Generally, good agreement was observed for the CTDIvol of the fan-beam CT (Table 3).

In Table 4, the shaded regions which compared “in-field” dose with CTDI for situations where the anatomy does not match the phantom used for the CTDI determination served as a test to assess the adequacy of utilizing CTDIvol as the sole metric for estimating relative changes in “in-field” imaging dose when selecting ClearRT image acquisition parameters. Although it would be expected for large differences in “in-field” and the provided CTDI to occur, it is still important for a clinician to have some estimate of the actual patient imaging dose. The data in Table 4 can provide an estimate of the degree of difference between CTDI and true patient dose for all three modalities when this situation arises. This is important especially for the “Whole Body” protocols because the CTDI was measured in the 36 cm diameter phantom but, as the name indicates, it is intended on being deployed for all anatomic sites. It is unclear why in Table 4 the instances of CBCT and fan-beam CT with the aforementioned mismatch between anatomy and CTDI phantom display a greater difference than ClearRT. Further investigation on that topic is needed.

In Table 4, the absolute difference in imaging “in-field” dose and the provided CTDI can be as much as 2 cGy per scan (Chin, Thorax, XL, Fine, 440 FoV). For a 25 to 35 fraction treatment course with daily image guidance, the additional dose could be 50–70 cGy. For radiation sensitive organs in the head/neck region, such as the spinal cord, this may be a factor to consider.

In Table 5, the out-of-field doses for ClearRT are greater for Coarse scan settings than for Fine scans. This relation is opposite for the “in-field” dose (refer to Table 1). For patients with highly radiation dose sensitive regions (e.g., pregnant patients, implanted pacemakers, etc.) just superior or inferior of the scan field, the Fine setting may be beneficial. It is also notable that the out-of-field dose is greater for ClearRT than CBCT. Conversely, with ClearRT the imaging region can be easily selected by the user but for CBCT, this cannot be changed as easily. These can be important factors to consider when choosing the best treatment modality for such clinical situations.

One of the main factors to consider when choosing an imaging protocol (other than the dose) is the image quality. Previous studies have shown that CNR has the widest variation when the tube current is varied [12] therefore, in this work, CNR is the focus for image quality. The CNR data are shown in Fig. 2. For reference, the CNR for fan-beam CT and CBCT systems are also included. The data in Fig. 2 may be useful in cases in which the trade-offs of low-contrast resolution must be balanced with the imaging dose. For example, in the case of using the “Head” protocol with a 440 FOV, one may consider using a “Normal” Mode setting with the “XL” Body Size parameter versus a “Fine” Mode with the “Medium” Body Size parameter which provides similar CNR. The vendor supplied CTDIvol for those two protocol settings are 1.9 and 1.6, respectively, so for similar contrast to noise ratios, the protocol with “Fine” Mode & “Medium” Body Size will deliver about 15% less imaging dose. Of course, another factor to consider is the imaging time; however, vendor software provides accurate scan times at the treatment console when a scan protocol is selected. Interestingly, less variation in the CNR is observed for the “Pelvis” scans, this could be due to the relatively greater mAs settings for the pelvis protocols than the others and the limited diameter of the CatPhan phantom.

Although calculated differently than in other works that prevent direct comparison, generally similar results were observed for the CNR. Velten et al. [12] compared “Fine” and “Coarse” scans to CBCT and a fan-beam CT and found that for many “Fine” scan protocols, ClearRT had greater CNR than fan-beam CT. The difference is that Velten et al. [12] found ClearRT to have the greatest CNR advantage for “Head” scans while we found the “Head” scans to deliver a lower CNR than the fan-beam CT. This is likely due to the features of different fan-beam CT scanners, although both are clinically employed CT simulators. Similarly, Velten et al. [12] found moderate to no improvement in CNR for “Coarse” scans. Tegtmeier et al. [13] also measured CNR as well as low-contrast visibility; a difference of interest is that the 270 FOV was found to have a lower CNR than a similar protocol utilizing a 440 FOV. This was not the case in the present study (see Fig. 2). The reason for this is unclear, but we estimate it is either from differences in defining CNR or due to changes in image reconstruction since Tegtmeier et al. [13] utilized a much earlier version of the system. Currently, our work is the only study to evaluate CNR using all the available Clear RT protocols.

For dose calculation accuracy, a stable relationship of CT number and density is required. Over a 27 month span the CT number to density table was updated twice. At month 10 (see Fig. 3), the clinically defined CT number threshold of ± 50 was slightly exceeded for the highest bone density material plug, which coincided with a software update. A second, larger, CT number change was observed at month 18, but this coincided with a software update and a power conditioner replacement. The change in CT number to density calibration was anticipated and recalibration was recommended by the vendor and communicated to the clinical physics staff ahead of the service work. Therefore, it is recommended to verify the CT number stability at vendor-recommended intervals as well as after service to the kV components.

## Conclusions

The CTDIvol measurements were compared to vendor-provided CTDIvol values for all three systems. Comparisons of the CTDIvol values between the systems were also performed. Considerable differences in “in-field” dose and CTDI was observed when the anatomy size was not congruent with the size of the phantom used for the vendor supplied CTDI which is expected, however, this work provides an estimate of the differences for a variety of ClearRT protocols. The CNR was measured for all combinations of ClearRT settings for comparisons within the system, as well as for a comparison with a CBCT and fan-beam CT system. The provided CNR and dose information can be clinically useful in selecting a proper ClearRT imaging protocol. Finally, the stability of CT number values for various tissue-equivalent materials was tracked over 27 months; two instances where the CT number constancy exceeded tolerance were observed, and both were observed after service.

## Funding statement

No funding was received for the conduct of this study

## Ethical compliance

This work does not include human subjects

## Data access statement

Data is not freely available at this time

## CRediT authorship contribution statement

**Eric D. Ehler:** Writing – review & editing, Writing – original draft, Visualization, Validation, Methodology, Investigation, Formal analysis, Data curation, Conceptualization. **Parham Alaei:** Writing – review & editing, Writing – original draft, Validation, Resources, Project administration, Methodology, Investigation, Formal analysis, Data curation, Conceptualization.

## Declaration of competing interest

The authors declare that they have no known competing financial interests or personal relationships that could have appeared to influence the work reported in this paper.
