# Supplementary material for: Imaging dose and image quality of kilovoltage imaging implemented on a helical tomotherapy unit
Source: Z Med Phys. 2025 Jan 21;36(1):60–70. doi: 10.1016/j.zemedi.2024.12.003 (PMC12901525; doi:10.1016/j.zemedi.2024.12.003)
Supplement: Supplementary Data 1 [file mmc1.docx]

| **Anatomy** | **Body Size** | **Mode** | **FOV** | **kV** | **mA/view** | **Filtration** | **Pitch** | **Slice Interval** | **Slice Thick.** | **Couch Speed** | **Views/Rotation** | **Beam Width** | **Gantry Period** | **mAs/rotation** |
| --- | --- | --- | --- | --- | --- | --- | --- | --- | --- | --- | --- | --- | --- | --- |
|  |  |  | (mm) |  |  |  |  | (mm) | (mm) | (mm/s) |  | (mm, at Iso) | (sec.) |  |
| **Head** | **Small** | **Fine** | **270** | 100 | 80 | 0.5 Cu | 0.86 | 1.2 | 2.4 | 5.24 | 600 | 50 | 8.22 | 240 |
|  |  |  | **440** |  |  | Bowtie | 0.75 |  |  | 4.57 |  |  |  |  |
|  |  | **Normal** | **270** |  |  | 0.5 Cu | 1.00 |  |  | 12.14 | 480 | 100 | 8.28 | 192 |
|  |  |  | **440** |  |  | Bowtie | 0.75 |  |  | 9.11 |  |  |  |  |
|  |  | **Coarse** | **270** |  |  | 0.5 Cu | 1.40 |  |  | 26.26 | 360 | 140 | 7.50 | 144 |
|  |  |  | **440** |  |  | Bowtie | 0.75 |  |  | 14.07 |  |  |  |  |
|  | **Medium** | **Fine** | **270** |  | 125 | 0.5 Cu | 0.86 |  |  | 5.24 | 600 | 50 | 8.22 | 375 |
|  |  |  | **440** |  |  | Bowtie | 0.75 |  |  | 4.57 |  |  |  |  |
|  |  | **Normal** | **270** |  |  | 0.5 Cu | 1.00 |  |  | 12.14 | 480 | 100 | 8.28 | 300 |
|  |  |  | **440** |  |  | Bowtie | 0.75 |  |  | 9.11 |  |  |  |  |
|  |  | **Coarse** | **270** |  |  | 0.5 Cu | 1.40 |  |  | 26.26 | 360 | 140 | 7.50 | 225 |
|  |  |  | **440** |  |  | Bowtie | 0.75 |  |  | 14.07 |  |  |  |  |
|  | **Large** | **Fine** | **270** |  | 160 | 0.5 Cu | 0.86 |  |  | 5.24 | 600 | 50 | 8.22 | 480 |
|  |  |  | **440** |  |  | Bowtie | 0.75 |  |  | 4.57 |  |  |  |  |
|  |  | **Normal** | **270** |  |  | 0.5 Cu | 1.00 |  |  | 12.14 | 480 | 100 | 8.28 | 384 |
|  |  |  | **440** |  |  | Bowtie | 0.75 |  |  | 9.11 |  |  |  |  |
|  |  | **Coarse** | **270** |  |  | 0.5 Cu | 1.40 |  |  | 26.26 | 360 | 140 | 7.50 | 288 |
|  |  |  | **440** |  |  | Bowtie | 0.75 |  |  | 14.07 |  |  |  |  |
|  | **X-Large** | **Fine** | **270** |  | 200 | 0.5 Cu | 0.86 |  |  | 5.24 | 600 | 50 | 8.22 | 480 |
|  |  |  | **440** |  |  | Bowtie | 0.75 |  |  | 4.57 |  |  |  |  |
|  |  | **Normal** | **270** |  |  | 0.5 Cu | 1.00 |  |  | 12.14 | 480 | 100 | 8.28 | 384 |
|  |  |  | **440** |  |  | Bowtie | 0.75 |  |  | 9.11 |  |  |  |  |
|  |  | **Coarse** | **270** |  |  | 0.5 Cu | 1.40 |  |  | 26.26 | 360 | 140 | 7.50 | 288 |
|  |  |  | **440** |  |  | Bowtie | 0.75 |  |  | 14.07 |  |  |  |  |
| **Thorax** | **Small** | **Fine** | **440** | 120 | 80 | Bowtie | 0.75 | 1.8 | 3.6 | 4.57 | 600 | 50 | 8.22 | 240 |
|  |  |  | **500** |  |  |  |  |  |  |  |  |  |  |  |
|  |  | **Normal** | **440** |  |  |  |  |  |  | 9.11 | 480 | 100 | 8.28 | 192 |
|  |  |  | **500** |  |  |  |  |  |  |  |  |  |  |  |
|  |  | **Coarse** | **440** |  |  |  |  |  |  | 14.07 | 360 | 140 | 7.50 | 144 |
|  |  |  | **500** |  |  |  |  |  |  | 13.41 |  | 134 |  |  |
|  | **Medium** | **Fine** | **440** |  | 125 |  |  |  |  | 4.57 | 600 | 50 | 8.22 | 375 |
|  |  |  | **500** |  |  |  |  |  |  |  |  |  |  |  |
|  |  | **Normal** | **440** |  |  |  |  |  |  | 9.11 | 480 | 100 | 8.28 | 300 |
|  |  |  | **500** |  |  |  |  |  |  |  |  |  |  |  |
|  |  | **Coarse** | **440** |  |  |  |  |  |  | 14.07 | 360 | 140 | 7.50 | 225 |
|  |  |  | **500** |  |  |  |  |  |  | 13.41 |  | 134 |  |  |
|  | **Large** | **Fine** | **440** |  | 160 |  |  |  |  | 4.57 | 600 | 50 | 8.22 | 480 |
|  |  |  | **500** |  |  |  |  |  |  |  |  |  |  |  |
|  |  | **Normal** | **440** |  |  |  |  |  |  | 9.11 | 480 | 100 | 8.28 | 384 |
|  |  |  | **500** |  |  |  |  |  |  |  |  |  |  |  |
|  |  | **Coarse** | **440** |  |  |  |  |  |  | 14.07 | 360 | 140 | 7.50 | 288 |
|  |  |  | **500** |  |  |  |  |  |  | 13.41 |  | 134 |  |  |
|  | **X-Large** | **Fine** | **440** |  | 200 |  |  |  |  | 4.57 | 600 | 50 | 8.22 | 600 |
|  |  |  | **500** |  |  |  |  |  |  |  |  |  |  |  |
|  |  | **Normal** | **440** |  |  |  |  |  |  | 9.11 | 480 | 100 | 8.28 | 480 |
|  |  |  | **500** |  |  |  |  |  |  |  |  |  |  |  |
|  |  | **Coarse** | **440** |  |  |  |  |  |  | 14.07 | 360 | 140 | 7.50 | 360 |
|  |  |  | **500** |  |  |  |  |  |  | 13.41 |  | 134 |  |  |
| **Pelvis** | **Small** | **Fine** | **440** | 140 | 80 | Bowtie | 0.75 | 1.8 | 3.6 | 4.57 | 600 | 50 | 8.22 | 240 |
|  |  |  | **500** |  |  |  |  |  |  |  |  |  |  |  |
|  |  | **Normal** | **440** |  |  |  |  |  |  | 9.11 | 480 | 100 | 8.28 | 192 |
|  |  |  | **500** |  |  |  |  |  |  |  |  |  |  |  |
|  |  | **Coarse** | **440** |  |  |  |  |  |  | 14.07 | 360 | 140 | 7.50 | 144 |
|  |  |  | **500** |  |  |  |  |  |  | 13.41 |  | 134 |  |  |
|  | **Medium** | **Fine** | **440** |  | 125 |  |  |  |  | 4.57 | 600 | 50 | 8.22 | 375 |
|  |  |  | **500** |  |  |  |  |  |  |  |  |  |  |  |
|  |  | **Normal** | **440** |  |  |  |  |  |  | 9.11 | 480 | 100 | 8.28 | 300 |
|  |  |  | **500** |  |  |  |  |  |  |  |  |  |  |  |
|  |  | **Coarse** | **440** |  |  |  |  |  |  | 14.07 | 360 | 140 | 7.50 | 225 |
|  |  |  | **500** |  |  |  |  |  |  | 13.41 |  | 134 |  |  |
|  | **Large** | **Fine** | **440** |  | 160 |  |  |  |  | 4.57 | 600 | 50 | 8.22 | 480 |
|  |  |  | **500** |  |  |  |  |  |  |  |  |  |  |  |
|  |  | **Normal** | **440** |  |  |  |  |  |  | 9.11 | 480 | 100 | 8.28 | 384 |
|  |  |  | **500** |  |  |  |  |  |  |  |  |  |  |  |
|  |  | **Coarse** | **440** |  |  |  |  |  |  | 14.07 | 360 | 140 | 7.50 | 288 |
|  |  |  | **500** |  |  |  |  |  |  | 13.41 |  | 134 |  |  |
|  | **X-Large** | **Fine** | **440** |  | 200 |  |  |  |  | 4.57 | 600 | 50 | 8.22 | 600 |
|  |  |  | **500** |  |  |  |  |  |  |  |  |  |  |  |
|  |  | **Normal** | **440** |  |  |  |  |  |  | 9.11 | 480 | 100 | 8.28 | 480 |
|  |  |  | **500** |  |  |  |  |  |  |  |  |  |  |  |
|  |  | **Coarse** | **440** |  |  |  |  |  |  | 14.07 | 360 | 140 | 7.50 | 360 |
|  |  |  | **500** |  |  |  |  |  |  | 13.41 |  | 134 |  |  |
| **Whole Body** | **Small** | **Fine** | **270** | 120 | 80 | 0.5 Cu | 0.86 | 3.6 | 3.6 | 5.24 | 600 | 50 | 8.22 | 240 |
|  |  |  | **440** |  |  | Bowtie | 0.75 |  |  | 4.57 |  |  |  |  |
|  |  |  | **500** |  |  |  |  |  |  |  |  |  |  |  |
|  |  | **Normal** | **270** |  |  | 0.5 Cu | 1.00 |  |  | 12.14 | 480 | 100 | 8.28 | 192 |
|  |  |  | **440** |  |  | Bowtie | 0.75 |  |  | 9.11 |  |  |  |  |
|  |  |  | **500** |  |  |  |  |  |  |  |  |  |  |  |
|  |  | **Coarse** | **270** |  |  | 0.5 Cu | 1.40 |  |  | 26.26 | 360 | 140 | 7.50 | 144 |
|  |  |  | **440** |  |  | Bowtie | 0.75 |  |  | 14.07 |  |  |  |  |
|  |  |  | **500** |  |  |  |  |  |  | 13.41 |  | 134 |  |  |
|  | **Medium** | **Fine** | **270** |  | 100 | 0.5 Cu | 0.86 |  |  | 5.24 | 600 | 50 | 8.22 | 300 |
|  |  |  | **440** |  |  | Bowtie | 0.75 |  |  | 4.57 |  |  |  |  |
|  |  |  | **500** |  |  |  |  |  |  |  |  |  |  |  |
|  |  | **Normal** | **270** |  |  | 0.5 Cu | 1.00 |  |  | 12.14 | 480 | 100 | 8.28 | 240 |
|  |  |  | **440** |  |  | Bowtie | 0.75 |  |  | 9.11 |  |  |  |  |
|  |  |  | **500** |  |  |  |  |  |  |  |  |  |  |  |
|  |  | **Coarse** | **270** |  |  | 0.5 Cu | 1.40 |  |  | 26.26 | 360 | 140 | 7.50 | 180 |
|  |  |  | **440** |  |  | Bowtie | 0.75 |  |  | 14.07 |  |  |  |  |
|  |  |  | **500** |  |  |  |  |  |  | 13.41 |  | 134 |  |  |
|  | **Large** | **Fine** | **270** |  | 125 | 0.5 Cu | 0.86 |  |  | 5.24 | 600 | 50 | 8.22 | 375 |
|  |  |  | **440** |  |  | Bowtie | 0.75 |  |  | 4.57 |  |  |  |  |
|  |  |  | **500** |  |  |  |  |  |  |  |  |  |  |  |
|  |  | **Normal** | **270** |  |  | 0.5 Cu | 1.00 |  |  | 12.14 | 480 | 100 | 8.28 | 300 |
|  |  |  | **440** |  |  | Bowtie | 0.75 |  |  | 9.11 |  |  |  |  |
|  |  |  | **500** |  |  |  |  |  |  |  |  |  |  |  |
|  |  | **Coarse** | **270** |  |  | 0.5 Cu | 1.40 |  |  | 26.26 | 360 | 140 | 7.50 | 275 |
|  |  |  | **440** |  |  | Bowtie | 0.75 |  |  | 14.07 |  |  |  |  |
|  |  |  | **500** |  |  |  |  |  |  | 13.41 |  | 134 |  |  |
